# Retrograde autobiographical amnesia following electroconvulsive therapy in patients treated for depression - a mixed-methods systematic review with meta-analysis and thematic meta-synthesis

**DOI:** 10.1192/j.eurpsy.2025.859

**Published:** 2025-08-26

**Authors:** A. Stachura, A. Butler, A. Borek, M. Chrétien, T. Sheth, H. Mithoowani, A. Kuc, H. Pham, A. Ćwiek, P. Jażdżyk, M. Wójcik

**Affiliations:** 1 2nd Department of Psychiatry, Institute of Psychiatry and Neurology, Warsaw, Poland; 2 Department for Continuing Education; 3 Nuffield Department of Primary Care Health Sciences, University of Oxford, Oxford, United Kingdom

## Abstract

**Introduction:**

Electroconvulsive therapy (ECT) is a treatment received by approximately 1.4 million people worldwide annually, depressive disorder being the most prevalent indication. Retrograde autobiographical amnesia (RAA) refers to difficulties in retrieving memories of past events. Despite being the most commonly reported side effect of ECT, its nature, duration and impact on patients’ lives remains uncertain.

**Objectives:**

(1) Assessing RAA severity in patients treated with ECT for depression compared with other treatment methods. (2) Assessing RAA severity in patients treated with right unilateral (RUL) vs bilateral (BL) ECT for depression. (3) Assessing overall RAA severity (pre-post effect) following an acute course of ECT. (4) Summarising patients’ lived experiences of RAA following ECT for depression.

**Methods:**

This systematic review was registered prospectively with PROSPERO (CRD42024445105). Seven databases were searched for eligible articles. Quantitative and qualitative studies assessing RAA in patients treated with ECT for depression published since 1985 were included. Abstract, full-text screening and data extraction were done in duplicates and independently. Quantitative data were meta-analysed using random effects model and qualitative data were analysed using thematic meta-synthesis.

**Results:**

Of initial 6126 records, 22 quantitaive and 20 qualitative studies were included. ECT caused significantly greater RAA compared with other treatments (SMD -0.73, 95% CI -1.31; -0.15, I2=54%, Figure 1). BL treatment caused significantly greater RAA than RUL (SMD -0.29, 95% CI -0.57; -0.01, I2=32%, Figure 2). The pre-post effects were big for RUL (SMD -0.77, 95% CI -1.15; -0.38, I2=93%, Figure 3) and BL ECT (SMD -1.16, 95% CI -1.79; -0.52, I2=93%). The main effect moderator was RAA assessment tool. Few studies reported delayed effects of ECT on RAA. Four analytical themes were identified from qualitative data: (1) Uncertainty regarding the cause, nature and severity of memory loss may cause distress for patients, undermine the quality of information provision and post-ECT care. (2) Ambiguous testimonies – perception of memory loss often shaped by ECT effectiveness. (3) Returning to ‘normal’ daily life may be a challenging, frustrating and lonely process, which requires developing adaptive coping strategies. (4) Some memories may not come back for years; re-leaning facts about oneself and reshaping own identity may be important steps on the journey to recovery.

**Image 1:**

**Figure:**
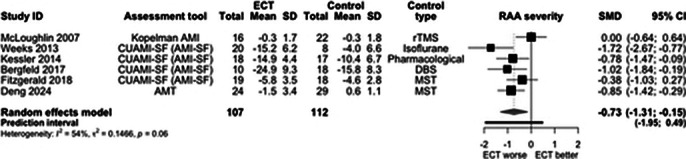

**Image 2:**

**Figure:**
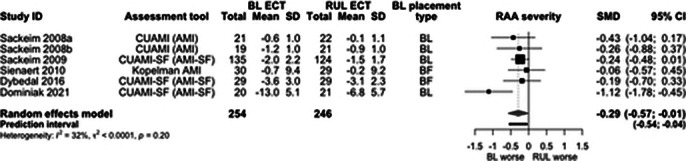

**Image 3:**

**Figure:**
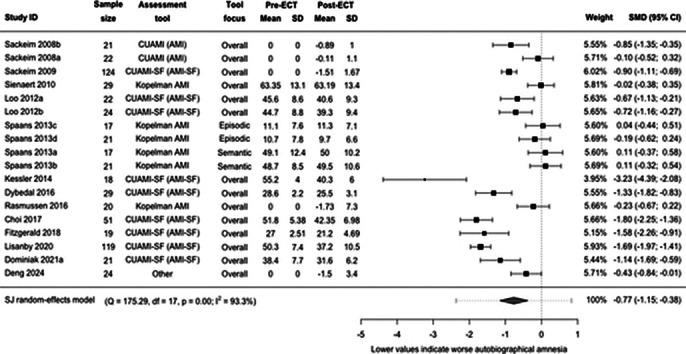

**Conclusions:**

ECT causes more severe (sometimes long-lasting) RAA than other forms of treatment with BL being more harmful than RUL. RAA measurement is not unified hindering identifying technical aspects of ECT, which may impact memory loss. Information provision and post-ECT care could be improved by reducing uncertainty around the nature and severity of RAA.

**Disclosure of Interest:**

None Declared

